# Predicting Student Performance Using Machine Learning in fNIRS Data

**DOI:** 10.3389/fnhum.2021.622224

**Published:** 2021-02-05

**Authors:** Amanda Yumi Ambriola Oku, João Ricardo Sato

**Affiliations:** Center of Mathematics, Computing and Cognition, Universidade Federal Do ABC, São Bernardo Do Campo, Brazil

**Keywords:** neuroscience, fNIRS, education, prefrontal cortex, machine learning, logistic regression, random forest

## Abstract

Increasing student involvement in classes has always been a challenge for teachers and school managers. In online learning, some interactivity mechanisms like quizzes are increasingly used to engage students during classes and tasks. However, there is a high demand for tools that evaluate the efficiency of these mechanisms. In order to distinguish between high and low levels of engagement in tasks, it is possible to monitor brain activity through functional near-infrared spectroscopy (fNIRS). The main advantages of this technique are portability, low cost, and a comfortable way for students to concentrate and perform their tasks. This setup provides more natural conditions for the experiments if compared to the other acquisition tools. In this study, we investigated levels of task involvement through the identification of correct and wrong answers of typical quizzes used in virtual environments. We collected data from the prefrontal cortex region (PFC) of 18 students while watching a video lecture. This data was modeled with supervised learning algorithms. We used random forests and penalized logistic regression to classify correct answers as a function of oxyhemoglobin and deoxyhemoglobin concentration. These models identify which regions best predict student performance. The random forest and penalized logistic regression (GLMNET with LASSO) obtained, respectively, 0.67 and 0.65 area of the ROC curve. Both models indicate that channels F4-F6 and AF3-AFz are the most relevant for the prediction. The statistical significance of these models was confirmed through cross-validation (leave-one-subject-out) and a permutation test. This methodology can be useful to better understand the teaching and learning processes in a video lecture and also provide improvements in the methodologies used in order to better adapt the presentation content.

## 1. Introduction

The interactivity in a virtual teaching environment can increase student engagement and, therefore, reinforces learned concepts and provide on-demand learning capacity (Jonassen et al., 1995). Empirical assessments have emerged in recent research, such as studies by Wachtler et al. (2018), which show that video lectures with quizzes can be used to increase knowledge, intensify engagement, and raise attention.

Although it is possible to measure student performance through the results of quizzes in class, a relevant factor to be studied is the involvement of students in the execution of tasks through the mapping of brain states during the task. Usually, cognitive neuroscience experiments study psychological processes through controlled manipulations, reducing the behavior of one of its components. However, this framework is not suitable when one wishes to generalize the characteristics of new situations from full descriptions of the behavior (Varoquaux and Poldrack, 2018). For instance, Barreto et al. (2020)and Noah et al. (2015) indicate the importance that studies involving music an dance be carried out under natural conditions. Similarly, Lamb et al. (2018) performs experiments under naturalistic conditions for the evaluation of science education.

We address this issue by performing an experiment in a more realistic setting. Specifically, we collected brain data with a fNIRS (functional near-infrared spectroscopy) device from students while they were watching a video lecture and answering questions. The fNIRS device was chosen due to its acquisition systems that collect data of hemodynamic states in several brain regions in a naturalistic, comfortable, and safe manner for participants (Noah et al., 2015). Safe levels of light (with wavelengths between 650 and 1,000 nm) were used to infer the variation in the level of oxygenation of brain tissue in a non-invasive way, which penetrates the biological tissue and reaches the cortex, allowing the analysis of oxygenation. hemoglobin (HbO_2_), deoxyhemoglobin (HHb) and total hemoglobin (tHb; tHb = (HbO_2_) + HHb) from cerebral blood (Delpy and Cope, 1997). The fNIRS technical limitations include superficial depth cortical evaluation (Ferrari et al., 2004). Specifically, we collected fNIRS data from the Prefrontal cortex (PFC).

The PFC has a central role in cognitive control. It has interconnections with brain areas that process external information (with all the sensory systems and structures of the cortical and subcortical motor system) and with internal information (limbic and midbrain structures involved in affection, memory, and reward). It has access and the means to influence processing in all major forebrain systems and can provide a means of synthesizing the various sources of information related to a given objective (Miller et al., 2002). McGuire and Botvinick (2010) shows there are indications that prefrontal cortex neurons appear to have a crucial ability for cognitive control, transmitting knowledge about a specific goal-directed task. Furthermore, Lamb et al. (2018) shows that fNIRS imaging of the prefrontal cortex can be useful to educators, since this region is responsible for problem solving, memory, and social behavior. However, this study also shows that tasks involving large amounts of unstructured processing, such as video lectures, can be challenging, since they generate less dynamic response within the prefrontal cortex than structured tasks.

In this paper, the fNIRS data from the PFC was used to create predictors for a student's answers. These predictors were obtained by applying machine learning algorithms to the data. In particular, we used random forests and penalized logistic regression algorithms. These algorithms allow one to understand the structure of existing data and generate prediction rules for new observations.

## 2. Materials and Methods

### 2.1. Participants

A total of 21 participants were recruited for participation but 3 of them were excluded (one for low signal quality and two for not meeting the health requirements). All 18 participants (10 female, 8 male) were right-handed, had normal vision and hearing, and mean age 25.6 ± 4.6 (range 18–40 years). No subject had an history of neurological or psychiatric disorders. Participants were recruited among undergraduate and graduate students in fields of Science. All participants alleged to have little or no prior knowledge in Astronomy. Signed consent was obtained from all members prior to participation. The Federal University of ABC - Ethics Committee approved the experiment. The experiment was performed in accordance with all local relevant guidelines and regulations. All subjects participated voluntarily and without any financial compensation, as required by federal laws.

### 2.2. Experiment

The experiment's tasks consisted of watching the first class in an Astronomy course while answering several multiple choice questions. The class was entitled “Astronomy: A general introduction”, and was chosen from a publicly available e-learning course from the Virtual University of São Paulo State (UNIVESP). The video's content usually does not belong to the basic education curriculum and requires reasoning and attention for understanding calculations and order of events. It was chosen since it brings new content to most students and does not require a large amount of previous knowledge.

Before running the main experiment, we tested the hypothesis that answering correctly depended on watching the video. This hypothesis was tested by applying the a quiz with multiple choice questions to a control group with 116 participants who did not watch the video lecture. The probability of a correct answer without watching the video was found based on a binomial test. The test did not reject the hypothesis that, without answering the video, participants answer correctly no better than by chance.

The main experiment was performed using Edpuzzle (http://edpuzzle.com/), an American platform for online learning. This platform was validated by Abou Afach et al. (2018) and is used by colleges, open courses, and universities. It was also validated in Brazil by researchers in education, which signaled it could be used successfully by local students (Lombardi and Gitahy, 2017).

We collected data of functional near-infrared spectroscopy (fNIRS) placed over the PFC (responsible for planning complex cognitive behavior, decision making, and moderating social behavior) of 18 undergraduate and graduate students using NIRSport equipment (company NIRx Medical Technologies). In the experiment, subjects were seated in a comfortable chair in a quiet and ventilated room.

**Figure 1:**
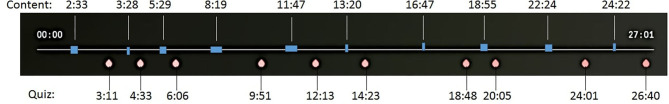
The questions are based on content exposed at earlier times throughout the video (indicated in blue). The red dots show the exact timing of the questions.

The subjects were asked to relax and to remain still during the experiment. They watched a free recorded lecture (27 min) with 10 multiple-choice exercises (Figure 1). As in real classroom situations, there was no indication of the times that they would be asked future questions.

### 2.3. Data Acquisition

**Figure 2:**
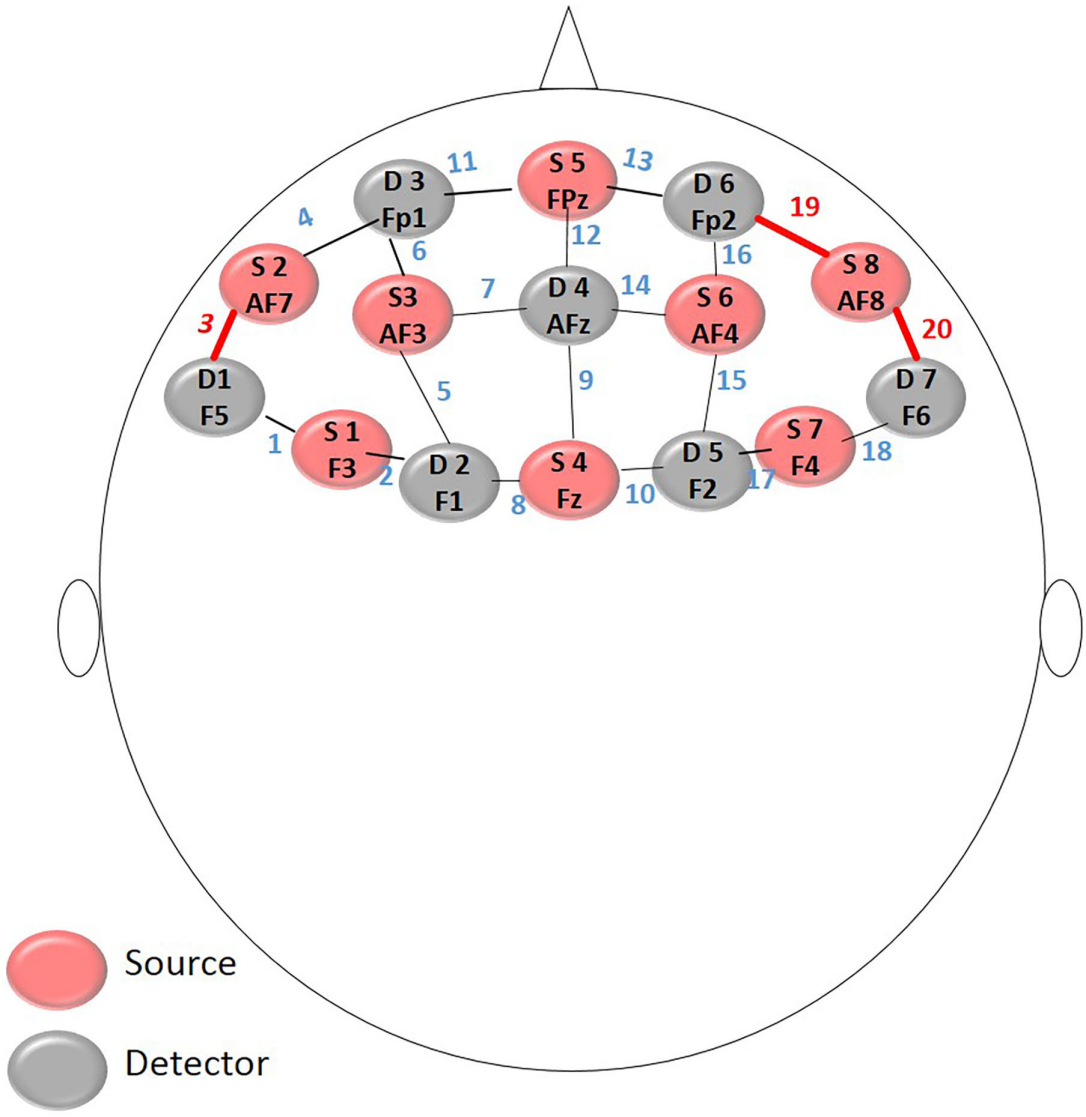
Montage layout: The position of the optodes follows the universal configuration of the 10-10.

The position of the optodes follows the universal configuration of the 10-10 electroencephalogram (EEG) system (Koessler et al., 2009). The 8 emitters and 7 detectors are positioned in the form: Sources in F3, AF7, AF3, Fz, Fpz, AF4, AF8, F4 and the Detectors in F5, F1, Fp1, AFz, F2, Fp2, F6 under an approximate distance of 3 cm between the optodes and resulting in the collection of oxyhemoglobin and deoxyhemoglobin from 20 channels, as Figure 2.

The recording of the PFC region was conducted on a multi-channel continuous wave system using NIRSport equipment (company NIRx Medical Technologies). This system consists of 8 illumination sources and 8 detection sensors with two wavelengths of 760–850 nm. The sampling rate of NIRSport is 62.5 Hz, as the device implements time multiplexing, which means that only one LED is turned on at each time, the sampling rate for each data channel is 7.81 Hz. The data were recorded by a computer during the measurements using NIRStar software (NIRx Medizintechnik GmbH, Berlin, Germany)

### 2.4. Data Preprocessing

Raw data from the NIRStar were processed using the NIRSLab-2014 (NIRx Medizintechnik GmbH,Berlin, Germany) via the Matlab 2007b (Mathworks, Natick, MA, USA) (Xu et al., 2014) software using a 0.01–0.2 Hz bandpass filter to reduce physiological signal artifacts at the cutoff frequencies of the global deviations (< 0.01 Hz), systemic interferences such as respiration rate (> 0.2*Hz*) and cardiac cycles (> 0.5*Hz*). We used the modified Beer-Lambert law (Mesquita and Covolan, 2008), to find the variations in oxygenated hemoglobin (HbO_2_) and deoxygenated hemoglobin (HHb) cited by Delpy and Cope (1997). We removed some motion artifacts manually (spikes) where HbO2 and HHb increased or decreased in unison based on visual inspection of the record (Lloyd-Fox et al., 2010). Afterward, we used the mean of the entire timeline as a baseline and differential path length factor (DPF) of 7.25 for the 760 nm and 6 wave, 38 for 850 nm lengths.

After computing the states of oxyhemoglobin (HbO_2_) and deoxyhemoglobin (HHb), the signal was averaged and grouped according to 10 exercises and 18 students, totaling 180 observations over 20 channels. The signal's standard deviation was also computed in each of these groups. However, since this feature did not improve the statistical analysis, it was not used in the final model.

### 2.5. Statistical Analysis

All learning algorithms were implemented in the R language (4.0.3 version). The “magrittr” and “tidyverse” packages were used in building the final database. The packages “randomForest” and “GLMNET” were used for fitting the Random Forest and Penalized Logistic Regression classifiers. Also, the “ROCR” package was used for performance analysis.

Logistic regression performs binary classification (dichotomous output labels), returning the probability that the object belongs to each class. In this way, the cost function can be the difference between the predicted probability and label 0 or 1. This cost can be estimated by calculating the average loss over all objects in a test set, similarly as done in linear regression.

Simple logistic regression can cause overfitting when dealing with many covariates. To mitigate this problem, we applied LASSO (least absolute shrinkage and selection operator) to our data. This is a regularization method that penalizes large parameter values and usually yields solutions in which the estimates of several of the parameters are zero (sparse solutions). This method is done by maximizing the log-likelihood added by a penalty factor. More details about LASSO can be found in section A.1 of the Appendix.

### 2.6. Cross Validation

Both our algorithms (Random Forest and GLMNET with LASSO) involved training 180-response BD (10 video ranges for each of the 18 subjects). Each of these has 40 covariates for prediction [mean (HbO_2_) and mean (HHb) for each of the 20 channels obtained in each video snippet].

Using a small database to learn the parameters of a prediction function and testing it on the same data can find a perfect score but would fail to predict yet-unseen data. This situation is called overfitting and can be overcome by cross-validation.

**Figure 3:**
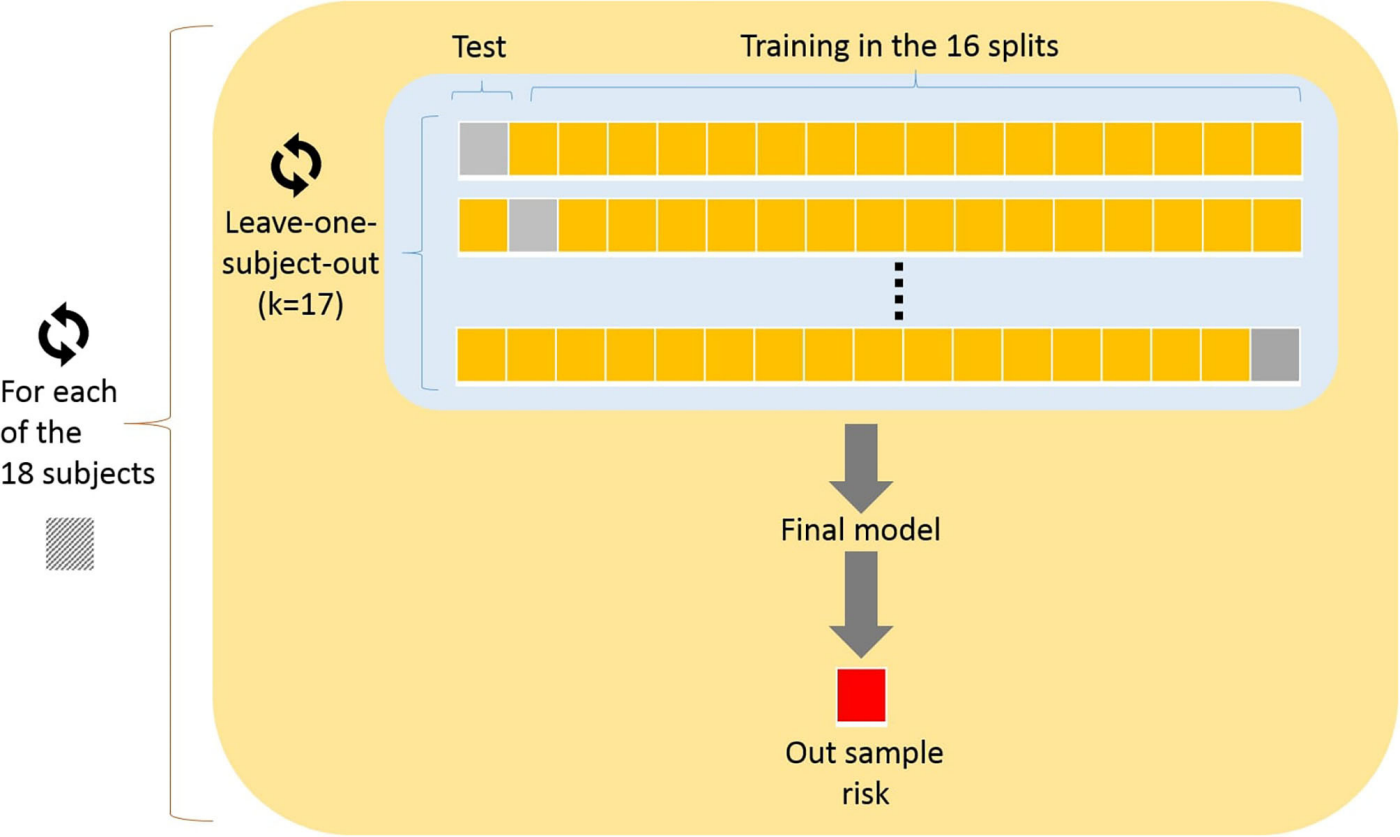
Double cross-validation implemented: In the outer (external) loop of double cross-validation, each interaction excludes one subject and all remaining data subjects are divided into two subsets referred to as training and test sets. The training set used in the inner (internal) loop, while the test set was exclusively used for model assessment.

The performance of Random Forest and LASSO logistic regression was evaluated using different types of cross-validation. The Random Forest was evaluated using simple leave-one-subject-out cross-validation. Also, we assessed the performance of LASSO logistic regression using double cross-validation (leave-one-subject-out) as illustrated in Figure 3. The double cross-validation process implemented comprises two nested cross-validation loops which are referred to as internal and external cross-validation loops. In the outer (external) loop of double cross-validation, each interaction excludes one subject and all remaining data subjects are divided into two subsets referred to as training and test sets. The training set used in the inner (internal) loop of double cross-validation for model building and model selection, while the test set was exclusively used for model assessment.

## 3. Results

**Figure 4:**
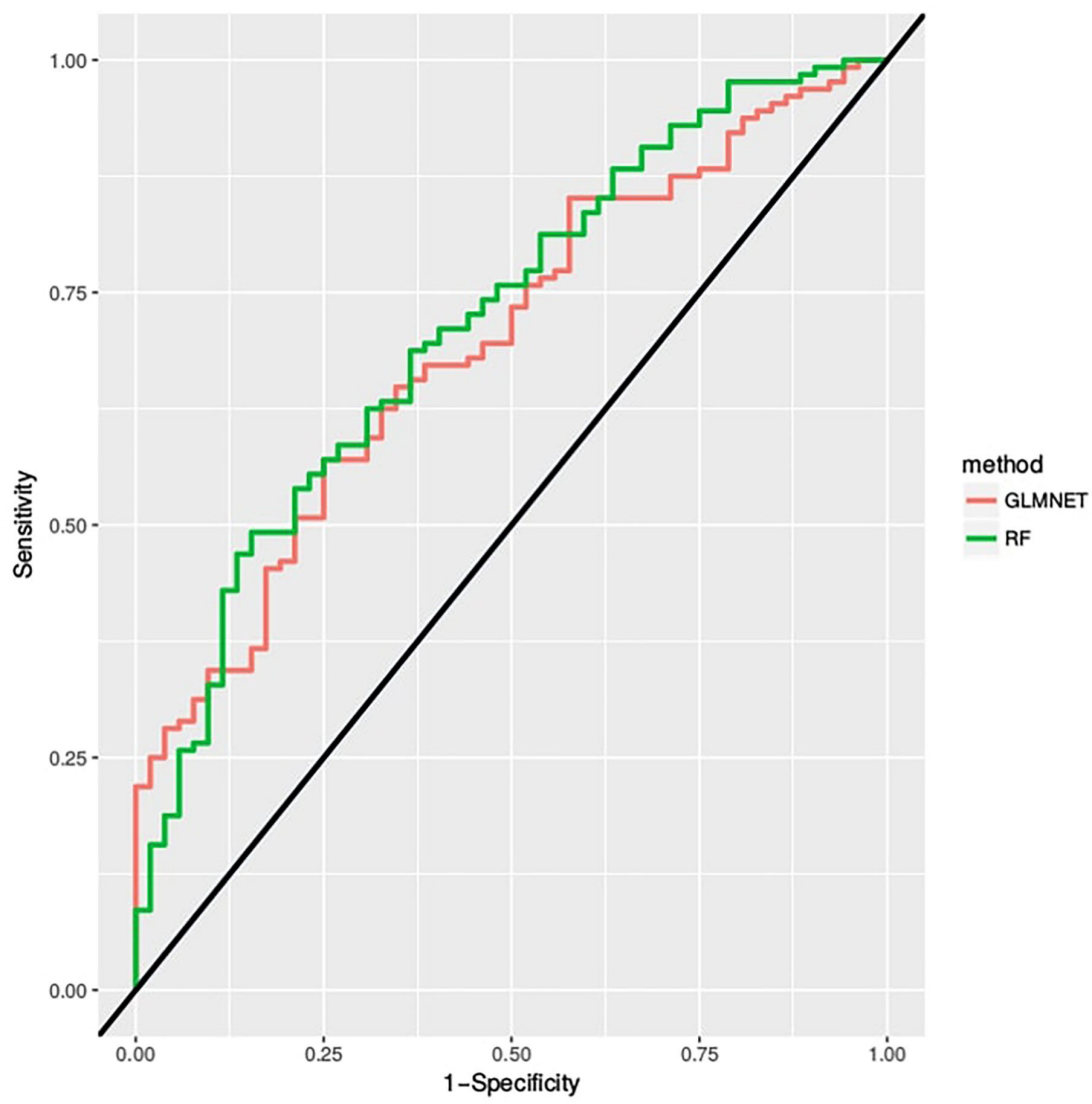
The ROC curve is created by plotting the true positive rate (sensitivity) against the false positive rate (specificity) at various threshold settings.

The Random Forest and the GLMNET obtained satisfactory results with, respectively, areas of 0.67 and 0.65 under the ROC curve in Figure 4. Also, We represented through the confusion matrix of both algorithms (Tables 1, 2) the instances of the predicted classes: Each row represents the instances of the predicted model while the column represents the real results of the students' performance. Both models obtained a good fit on identifying actual right answers (correct/correct) and wrong answers (incorrect/incorrect). The GLMNET LASSO had an accuracy of 0.63 ± 0.036, a sensitivity of 0.62 ± 0.067, a specificity of 0.64 ± 0.042, and a Cohen's kappa coefficient of 0.22 (fair on the Kappa scale). The random forest had a slightly better result, with an accuracy of 0.66 ± 0.035, a sensitivity of 0.63 ± 0.066, a specificity of 0.66 ± 0.042, and a Cohen's kappa coefficient of 0.26 (fair on the Kappa scale).

**Table 1:** Confusion matrix—random forest.

**Predicted \Actual**	**Incorrect**	**Correct**
Incorrect	34	44
Correct	18	84

**Table 2:** Confusion matrix—GLMNET.

**Predicted \Actual**	**Incorrect**	**Correct**
Incorrect	32	46
Correct	20	82

We also showed that the models are in fact better than chance through a permutation test, which evaluates whether the model is uninformative. This test can be easily applied to a wide range of statistical learning methods, including some in which a measure of variability is difficult to obtain and is not automatically produced by the statistical software (Friedman et al., 2001).

We repeated the same procedure of adjusting the models with the shuffled response variables and calculated the AUC (area under the ROC curve) for each one of the 1, 000 iterations.The total number of cases that resulted in a better model than the original was 3 cases for the Random Forest, thus obtaining a *p*-value of 0.003 (thus rejecting the null hypothesis) and the total number of cases that resulted in a better model than the original was 1 in GLMNET, thus obtaining a p-value of 0.001 (also rejecting the null hypothesis).

The output of the models identified which channels resulted in better predictors for the exercises.

### 3.1. Main Predictors—Penalized Logistic Regression

**Figure 5:**
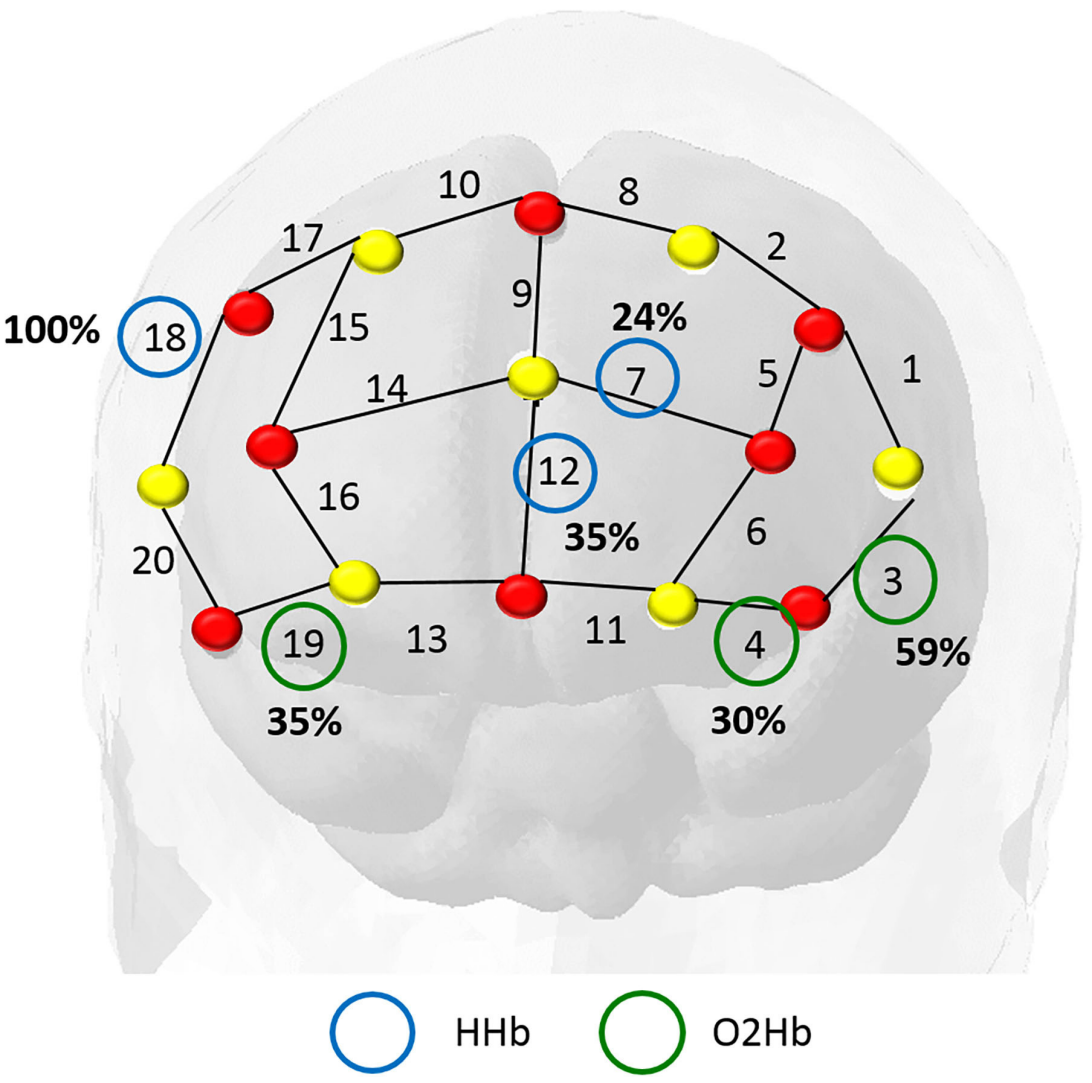
In this map, the red dots represent the sources and the yellow dots the detectors. We identified the most important channels from the total iterations in training the model. The frequency of the main covariables identified were: deoxyhemoglobin (blue circles) in channel 18 (highly relevant in all subjects) and oxyhemoglobin (green circles) in channel 3 (present in 60% of the subjects).

For the GLMNET model, we calculated the frequency of the selected channels in each iteration of the outer loop of the cross-validation, as displayed in Figure 5. We verified that the covariates (HHb) in channel 18 (referring to regions F4-F6 in the 10-10 system) and the (HbO_2_) in channel 3 (F5-AF7) had greater weight in the prediction, being used in, respectively, 100 and 59% of the subjects.

The relevant channels according to this model are the areas of channel 4 (AF7- F5), and channel 18, regions F4-F6, both corresponding to middle frontal cortex (Koessler et al., 2009; Balconi and Fronda, 2020). The region belongs to the dorsolateral prefrontal cortex (Bandeira et al., 2019) which is associated with the cognitive process, working memory, cognitive flexibility, planning, inhibition, and abstract reasoning (Zgaljardic et al., 2010).

As for the most important channels for each of the models, it is worth mentioning that the penalty of the channels in the GLMNET with LASSO does not imply that they are not explanatory for the response variable, but rather, there may be a correlation with another channel that is explanatory and therefore suffered a penalty.

### 3.2. Main Predictors—Random Forest

**Figure 6:**
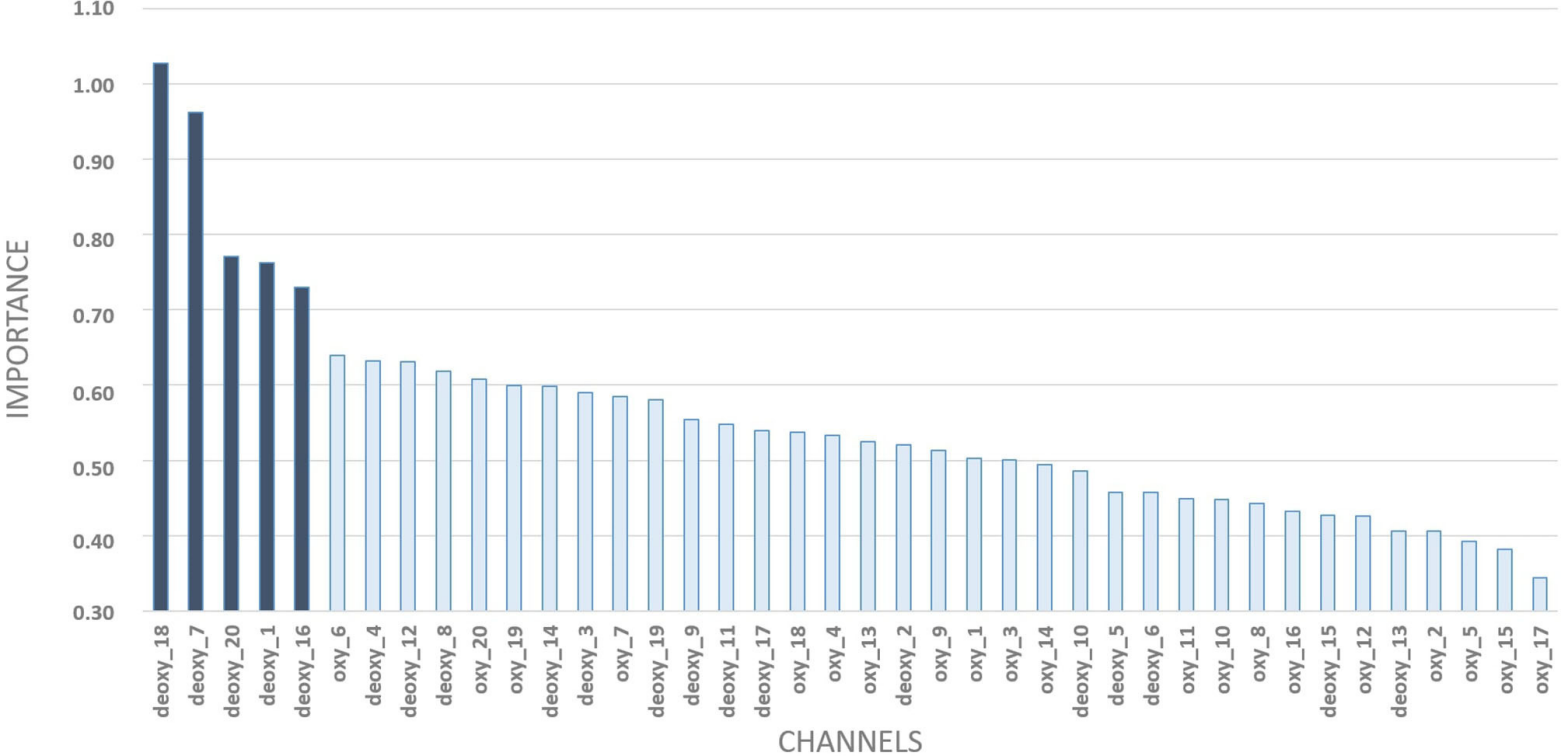
Random forest outputs: level of importance of each covariate with a detailed zoom at the top-5 ones.

The Random Forest Model indicated high predictive power from the covariates (HHb) in channel 18 (Figure 6). Besides this channel, the following were the most relevant: 7 (AF3-AFz), 20 (AF8-F6), 1 (F3 and F5), and 16 (AF4 and Fp2). In addition to working memory, they also show semantic aspects of language.

### 3.3. Hits Expectations vs. Prediction

In addition to identifying which channels are more explanatory for identifying the errors in the questions per individual, it was also possible to evaluate the levels of student involvement in interactive classes. We analyzed which types of questions are more difficult to answer by comparing the error rate with moments when the students declared to have lost concentration.

**Figure 7:**
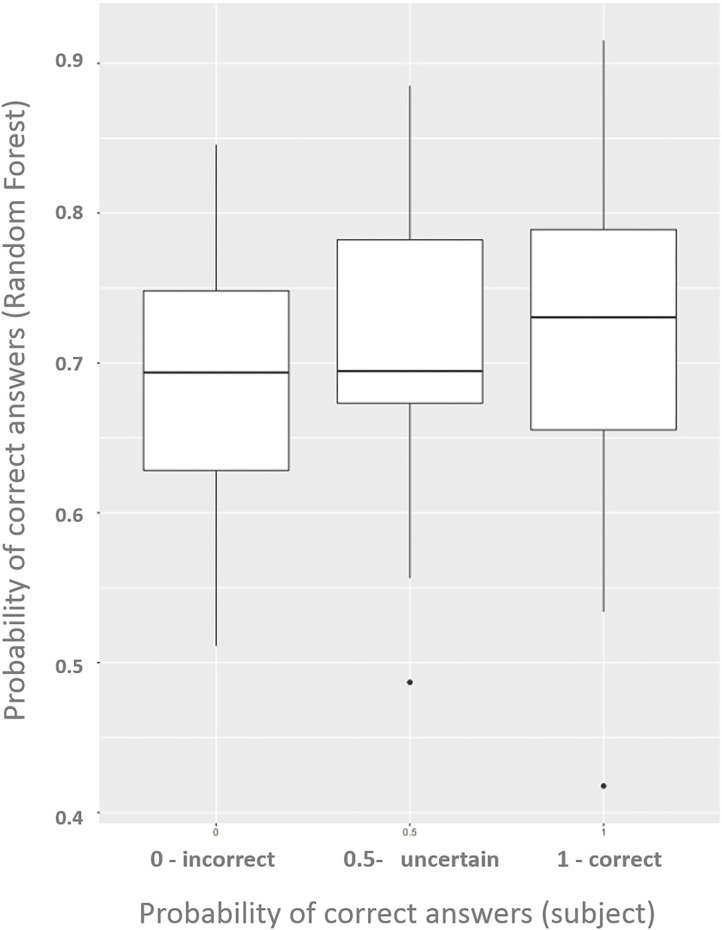
Boxplots show differences between the groups: 1, certainly right exercise; 0.5, not sure/next idea; 0, probably wrong/random guess.

We compared the results of the random forest prediction with what the volunteers believed they had got right and mentioned at the end of the experiment. The Figure 7 shows how the model differentiates hits and errors using only signals of oxyhemoglobin and deoxyhemoglobin in each question.

The Random Forest model indicates a slightly higher probability of correct answers among the cases in which the subjects believe they have hit the exercise. Also, it indicates a low probability of correct answers for the cases in which the subjects declared to have felt indecisive or believed to have mistaken the question (in this case, with low differentiation between them).

For the training of the models, it was necessary to identify the hemodynamic signs linked to the questions. We conducted tests to assure that the questions alone were not enough to predict student successes and errors (which would show an error in the design of the experiment).

The analyzed regions of the experiment are only suitable for exercises with the fixation of theoretical content. Mathematical reasoning, calculation, and perception have not been validated.

## 4. Discussion

In this research, we fit a predictive model for a students' correctness of answers in an interactive class based on PFC activity. These models allowed the identification of which regions are most relevant and influence results the most.

**Figure 8:**
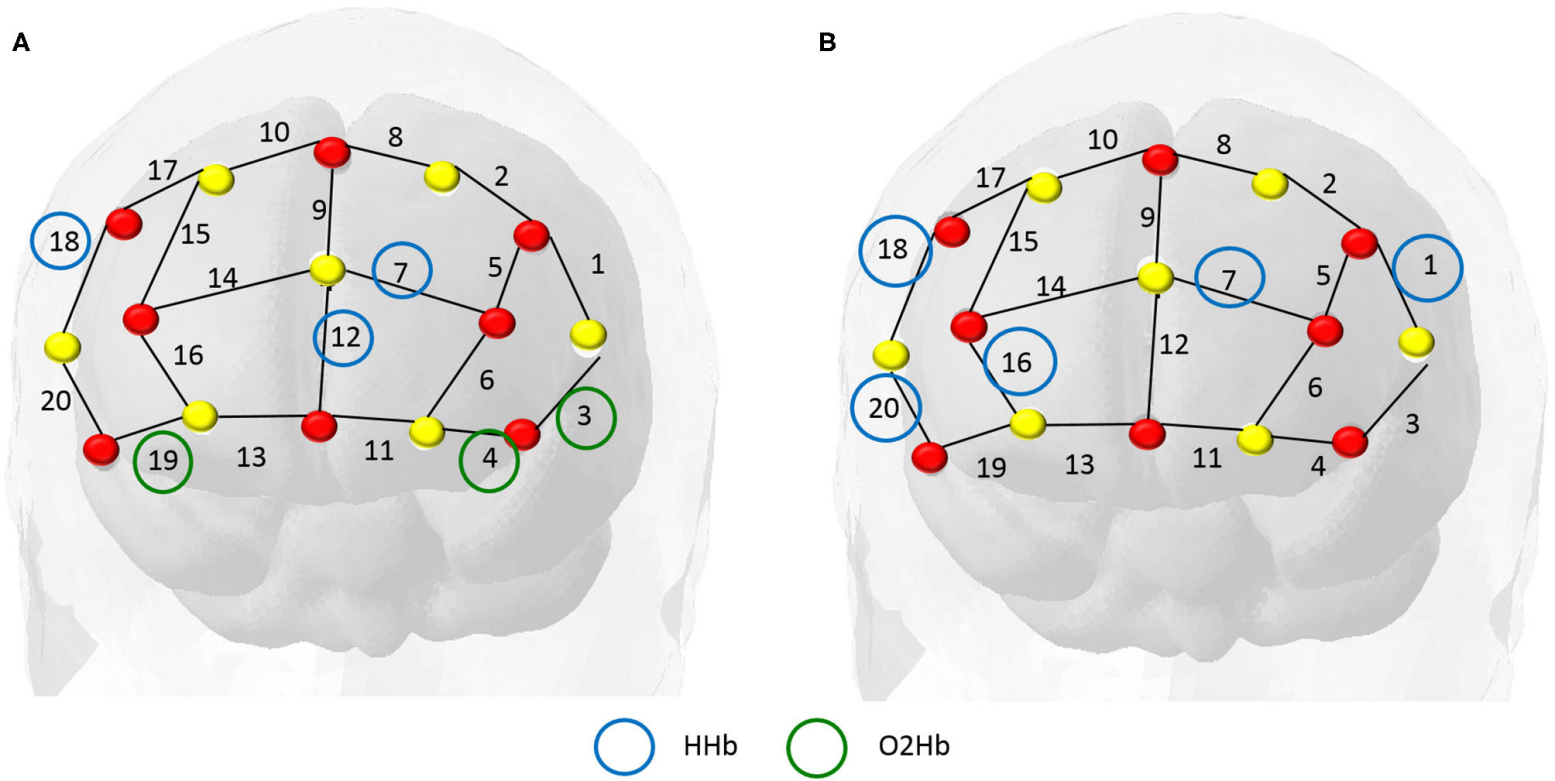
In these maps, the red dots represent the sources and the yellow dots the detectors. Panel **(A)** refers to the GLMNET Model output and strongly indicates channel 18 HHb (F4-F6) and channel 4 O2Hb (AF7-FP1). Panel **(B)** refers to the Random Forest Model output and indicates greater relevance for channel 18 HHb (F4-F6) and channel 7 HHb (AF3- AFz). The channel 18 region is the dorsolateral prefrontal region, associated with attention and working memory.

Both models (Figure 8) indicated that the information from channels F4-F6 (based on the EEG 10-10 system) had the greatest impact on the predictive model (Figure 4), suggesting a significant contribution to language understanding and semantic decision tasks.

Our models are consistent with other articles in the literature. For instance, (Liu and Ayaz, 2018) shows that perceived speech can be identified from the listeners' brain signals measured with fNIRS and (Herff et al., 2014) shows that measuring hemodynamic responses in the PFC with fNIRS, they showed the degree of workload a subject was experiencing, instead of only identify if there was an engagement during the tasks. Furthermore, MacDonald et al. (2000) and Dosenbach et al. (2006) use fNIRS data to show that brain activity can distinguish between high and low levels of task engagement. Specifically, they detected differences in the brain activity in the dorsolateral prefrontal cortex (*dorsolateral prefrontal cortex*—DLPFC) while participants alternated between performing and not performing a cognitive task.

With error rates in the models below 30%, our work can be suggested to assess levels of student involvement in tasks to validate new teaching content through videos, allowing us to evaluate whether students can assimilate content from fNIRS signals.

Despite the results obtained, the study has some limitations. For instance, the model considers the NIRS signal related to a single video lesson. Further studies are needed to have more information about students' behavior and performance during the task. Also, in the collection of fNIRS data in this experiment, we did not use short distance detectors, which could assist in the exclusion of extracerebral signals around the sources (Tachtsidis and Scholkmann, 2016).

An unexpected result was the high importance of HHb in both predictive models. Usually fNIRS studies indicate a high influence of HHbO_2_ on results, with higher signal-to-noise ratio SNR than HHb. Fishburn et al. (2014) shows the fNIRS sensitivity to detect linear changes in activation and functional connectivity in response to cognitive load, using HHbO_2_ and HHb had low correspondence. Also, Fishburn et al. (2014), Leon-Dominguez et al. (2014), and Barreto et al. (2020) show significant results for HHb. The sensitivity and SNR are core parameters during the fNIRS measurement and from the results obtained, further investigation is needed regarding the importance of HHb data in the models and new systematic analysis of SNR.

Since our primary goal was limited to investigating the PFC, we did not acquire signals from other brain regions. Although this assembly of optodes provides favorable conditions for more realistic situations, complementary studies with Functional Magnetic Resonance Imaging (fMRI) could perform to accurately identify other brain regions and also identify a precise location of Brodmann's areas involved during the task.

This study opens perspectives for a better understanding of the PFC during the execution of tasks and experiments in real situations. For further studies, we understand that it is important to continue assessing the level of sustained attention of students from hemodynamic states through models for classifying the involvement in the task rather than subtasking specific tasks.

## Data Availability Statement

The raw data supporting the conclusions of this article will be made available by the authors, without undue reservation.

## Ethics Statement

The studies involving human participants were reviewed and approved by Federal University of ABC - Ethics Committee approved all this experiment. The experiment was performed in accordance with all local relevant guidelines and regulations. The patients/participants provided their written informed consent to participate in this study.

## Author Contributions

AO and JS: designed the study, collected, and analyzed the data and revised the manuscript. All authors have read and agreed to the published version of the manuscript.

## Conflict of Interest

The authors declare that the research was conducted in the absence of any commercial or financial relationships that could be construed as a potential conflict of interest.

## A. Appendix

### A.1. LASSO

Logistic regression is a supervised learning method that is used for binary response variables. Let *Y*_*i*_ ∈ {0, 1} be a response variable and 𝕏_*i*_ be a vector of covariates. In logistic regression, the logit of ℙ(*Y*_*i*_ = 1|𝕏_*i*_) follows a linear equation, that is,

(A1) $$\begin{array}{ll} \text{log}\left(\frac{{\text{ℙ}}_{\beta }\left(Y_{i}=1|{\text{𝕏}}_{i}\right)}{1-{\text{ℙ}}_{\beta }\left(Y_{i}=1|{\text{𝕏}}_{i}\right)}\right)=\beta^{t}{\text{𝕏}}_{i} & \;,\text{where }\beta \text{ are}\\ \text{ unknown coefficients.} \end{array}$$

Using Equation (1), it is possible to compute the log-likelihood of coefficients, *l*(*β*), for the observed sample.

(A2) $$\begin{array}{l} l\left(\beta_{0}\right)=\overset{n}{\underset{i=1}{\sum }}\text{log}\left({\text{ℙ}}_{\beta_{0}}\left(Y_{i}=y_{i}|{\text{𝕏}}_{i}\right)\right) \end{array}$$

The value of *l*(β_0_) is a measure of how likely it is that β = β_0_. Based on this interpretation, a common choice of estimator for β is the one which maximizes *l*(β_0_), the maximum likelihood estimator. However, this estimator can lead to overfitting when the sample size is small relatively to the number of covariates. In this case, it is common to use regularized maximum likelihood estimators.

LASSO is one alternative for performing regularized logistic regression. In this framework, one estimates *β* by maximizing

(A3) $$\begin{array}{l} s\left(\beta_{0}\right)=l\left(\beta_{0}\right)-\lambda \overset{d}{\underset{i=1}{\sum }}|\beta_{i}|. \end{array}$$

Equation 3 leads to a trade-off between how likely is *β* and how small are its values. This trade-off often avoids overfitting and leads to better estimators. Furthermore, in LASSO one uses a *l*_1_ penalty, $\overset{d}{\underset{i=1}{\sum }}|\beta_{i}|$. This penalty often leads to estimates for *β* that have many zeroes. That is, LASSO estimation often automatically performs feature selection.
